# Complete genome sequence of a hydrogenotrophic methanogen, *Methanobacterium* sp. ACI-7, isolated from an alkaline–saline wetland

**DOI:** 10.1128/mra.00213-25

**Published:** 2025-08-25

**Authors:** Nicole A. Fiore, Karrie A. Weber

**Affiliations:** 1 School of Biological Sciences, University of Nebraska-Lincoln 14719, Lincoln, Nebraska, USA; 2 Department of Earth and Atmospheric Sciences, University of Nebraska-Lincoln 14719, Lincoln, Nebraska, USA; 3 Daugherty Water for Food Institute, University of Nebraska, Lincoln, Nebraska, USA; Indiana University, Bloomington, Bloomington, Indiana, USA

**Keywords:** methanogen, methanogenesis, archaea, genome, *Methanobacterium*, methane, hydrogenotrophy, hydrogen

## Abstract

*Methanobacterium* sp. ACI-7 was isolated from an enrichment culture established with calcium carbonate as the sole inorganic carbon source originating from alkaline–saline wetland soil. The genome sequence consisted of a single, circular 2,239,949 bp chromosome. A total of 2,366 protein-coding genes, 37 tRNAs, and 6 rRNAs were predicted.

## ANNOUNCEMENT

Hydrogenotrophic methanogens are archaea that convert hydrogen and inorganic carbon to methane, influencing both the global carbon cycle and climate (1). Here, we report the genome sequence of the hydrogenotrophic methanogen *Methanobacterium* sp. strain ACI-7, recently isolated from a previously described (2) enrichment culture (C2M) initiated from alkaline–saline wetland soil with calcium carbonate as a sole source of inorganic carbon. Prior to isolation, the methanogen was further enriched from the C2M community using MS Enrichment (MSE) medium (3) supplemented with antibiotics (100 µg/mL kanamycin, 100 µg/mL clindamycin, 100 µg/mL tetracycline, and 250 µg/mL vancomycin). Hydrogen gas was added to the headspace after inoculation, and cultures were incubated horizontally at 37°C. Single colonies were then isolated from 1.7% (wt/vol) agar shake tubes (4) of MSE medium in an anaerobic chamber. All subsequent cultivation was performed using liquid MSE medium without antibiotics. One pure culture, designated ACI-7, was selected for whole genome sequencing based on measured methane production.

For the extraction of genomic DNA, 50 mL of liquid culture (mid-log phase) was collected on a sterile, 0.2 µm, polycarbonate filter membrane and flash frozen in liquid nitrogen. Cells were lysed by grinding with a mortar and pestle in liquid nitrogen and by heating (65°C) for 1 h in a high-salt extraction buffer containing 1% hexadecylmethylammonium bromide and 2% sodium dodecyl sulfate (5). Genomic DNA was purified from the lysate with phenol:chloroform:isoamyl alcohol and chloroform:isoamyl alcohol followed by ethanol precipitation (6). Library preparation and sequencing were completed by the University of Delaware DNA Sequencing and Genotyping Center. Approximately 1 µg of genomic DNA was used for library preparation (PacBio SMRTbell prep kit 3.0), followed by size selection (BluePippin) from 6 kB. Sequencing was conducted with the PacBio Sequel IIe system, which generated a total of 11,788,604,596 bases comprising 1,063,330 polymerase reads, with a mean read length of 11,086 nucleotides. Reads were assembled with Flye 2.9.1 (7) (--pacbio-hifi and --asm-coverage 100), resulting in a single, circular contig 2,239,949 bp in length with 31.79% GC content. Read mapping and coverage were calculated using SAMtools 1.20 (8), with 100% of reads mapping to the assembly at an estimated 5253× coverage.

Genome annotation was performed with the NCBI Prokaryotic Genome Annotation Pipeline 6.8 (9), which predicted 2,411 genes representing 2,360 protein-coding sequences, 2 complete sets of rRNAs (5S, 16S, and 23S rRNAs), 37 tRNAs, and 2 noncoding RNAs (ncRNAs). Genes were identified for all enzymes associated with the Wolfe cycle of methanogenesis (10), including formylmethanofuran dehydrogenase, formylmethanofuran-tetrahydromethanopterin N-formyltransferase, methenyltetrahydromethanopterin cyclohydrolase, methylenetetrahydromethanopterin dehydrogenase, 5,10-methylenetetrahydromethanopterin reductase, tetrahydromethanopterin S-methyltransferase, methyl-coenzyme M reductase, heterodisulfide reductase, and coenzyme F420 hydrogenase. Genome completeness and contamination were determined with CheckM v1.1.2 (11) using the “lineage_wf” option. Based on conserved single-copy genes (p__Euryarchaeota), the genome is 99.2% complete with no contamination. Taxonomic classification with GTDB-Tk v2.4.0 (12) assigned the ACI-7 genome to the genus *Methanobacterium*. A summary of sequencing metrics and genomic characteristics of *Methanobacterium* strain ACI-7 is provided in Table 1.

**TABLE 1:** Genome sequencing, assembly, and annotation data for *Methanobacterium* sp. ACI-7

Feature	Value
Genome characteristics	
Genome size (bp)	2,239,949
Coverage	5253×
G + C content (%)	31.79
Contigs	1
Circular	Yes
Completeness	99.2
Contamination	0.0
No. of genes	2,411
No. of coding sequences	2,366
No. of rRNAs (5S, 16S, 23S)	6 (2, 2, 2)
No. of tRNAs	37
Sequencing metrics	
Bases	11,788,604,596
Polymerase reads	1,063,330
Mean read length (bp)	11,086
Data availability	
BioProject	PRJNA1143933
GenBank accession no.	CP166866
Sequence Read Archive accession no.	SRR30209458

## Data Availability

This genome project has been deposited with the National Center for Biotechnology Information (NCBI) under the BioProject accession number PRJNA1143933. The complete genome sequence for *Methanobacterium* sp. ACI-7 can be found under the GenBank accession number CP166866. Raw reads are available via the Sequence Read Archive (SRA) using the accession number SRR30209458.
